# Hemangiosarcoma in dogs as a potential non-rodent animal model for drug discovery research of angiosarcoma in humans

**DOI:** 10.3389/fonc.2023.1250766

**Published:** 2023-12-07

**Authors:** Kazuki Heishima, Naohiko Aketa, Mizuki Heishima, Asuka Kawachi

**Affiliations:** ^1^ Institute for Advanced Study (GUiAS), Gifu University, Gifu, Japan; ^2^ Center for One Medicine Innovative Translational Research (COMIT), Gifu University, Gifu, Japan; ^3^ Clinical and Translational Research Center, Keio University Hospital, Tokyo, Japan; ^4^ IDEXX Laboratories, Tokyo, Japan; ^5^ Division of Cancer RNA Research, National Cancer Center, Tokyo, Japan; ^6^ Department of Medical Oncology, National Cancer Center Hospital, Tokyo, Japan

**Keywords:** angiosarcoma, hemangiosarcoma, cancer heterogeneity, non-conventional animal model, drug discovery

## Abstract

Since the domestication of dogs 10,000 years ago, they have shared their living environment with humans and have co-evolved. The breeding process that dogs have undergone in only a few centuries has led to a significant accumulation of specific genetic alterations that could induce particular diseases in certain breeds. These canine diseases are similar to what is found in humans with several differences; therefore, comparing such diseases occurring in humans and dogs can help discover novel disease mechanisms, pathways, and causal genetic factors. Human angiosarcoma (AS) and canine hemangiosarcoma (HSA), which are sarcomas originating from endothelium, are examples of diseases shared between humans and dogs. They exhibit similar characteristics and clinical behaviors, although with some critical differences resulting from evolution. In this review, we will describe the similarities and differences in terms of clinical and molecular characteristics between human AS and canine HSA, and discuss how these similarities and differences can be applied to advance the treatment of these diseases.

## Introduction

Dogs were domesticated more than 10,000 years ago in southern East Asia (1–4). Since then, humans and dogs have co-evolved in a shared living environment, exposed to the same pathological and dietary conditions (5). Dogs have undergone unique evolutionary changes through selective breeding, resulting in a diverse range of breeds with variations in their morphology, physiology, and behavior. However, these processes have also led to a significant accumulation of genetic alterations and specific diseases in certain breeds (6, 7). Many of the diseases closely resemble disorders that affect humans with several minor differences; therefore, comparing such diseases occurring in humans and dogs can help discover novel disease mechanisms, pathways, and causal genetic factors. Indeed, this approach has successfully contributed to the discovery of novel disease mechanisms, pathways, and causal genetic factors of human diseases, including *NFAT* in SLE-like disease (8), *HAS2* biosynthesis in autoinflammatory disease (9), *LGI2* in remitting focal epilepsy (10), and *SLC4A3* in progressive retinal atrophy (11).

Representative examples of diseases shared between humans and dogs include sarcomas with vascular origins, such as human angiosarcoma (AS) and canine hemangiosarcoma (HSA) (12–14). Both human AS and canine HSA are highly aggressive sarcomas derived from vascular-forming cells, with limited treatment options and high mortality rates. They share many disease characteristics, including molecular profiles and treatment responses. However, they also exhibit critical differences in their incidence rates. Human AS is a rare cancer, accounting for approximately 0.01% of all cancers (12, 14). The rarity of human AS has hindered the development of new therapeutics and biomarkers, despite a significant unmet medical need for new diagnostics and therapies for AS patients. Even basic research tools for AS, such as cell lines and mouse models, are limited. Canine HSA, on the other hand, has an extraordinarily high incidence rate in specific dog breeds (13). The high incidence rate in dogs offers numerous advantages for investigating the clinical responses to therapeutics and the basic biology of the disease, given its clinical and genetic similarities to human AS. Therefore, canine HSA may serve as a unique model for drug discovery research aimed at providing a new treatment option to improve the prognosis of human AS. In this context, we describe the characteristics of canine HSA and discuss how their similarities and differences can be applied to advance the treatment of these diseases.

## Classification and general prognosis

Different pathological terminologies have been employed for human AS and canine HSA, which are further differently subclassified based on the disease characteristics of the primary tumor site or etiology.

Human AS encompasses multiple types of endothelial cell-derived sarcomas; i.e., sarcomas derived from endothelial cells of blood vessels (HSA) and lymphatic vessels (lymphangiosarcoma). AS is typically subclassified based on the primary tumor site (cutaneous, soft-tissue, breast, and visceral AS) or etiology (lymphedema-associated and radiation-induced AS) (15–17). Each AS subtype has different prognoses and disease courses. Generally, localized cutaneous AS has a relatively favorable prognosis with 2-year overall survival rates (OS) of 71.6 to 94.1% (18); however, metastatic AS has a poor prognosis with a median OS of 8 to 9.9 months (12, 19).

The canine counterparts of human AS are still referred to separately as HSA or lymphangiosarcoma. HSA is the predominantly reported subtype, while only a small number of cases have been reported as lymphangiosarcoma (20, 21). However, it should be noted that the majority of reports use diagnostic markers such as CD34, CD31, and Factor VIII-related antigen (F8RA), which cannot differentiate between HSA and lymphangiosarcoma. Lymphatic vessel markers like LYVE-1 and PROX-1 are rarely used in veterinary medicine to exclude the possibility of lymphangiosarcoma (22). Although evidence of tumor-associated vessels containing blood cells may help to distinguish HSA from lymphangiosarcoma, it would not completely exclude the possibility of the lesion being lymphangiosarcoma. Therefore, a certain number of cases classified as HSA in veterinary medicine may have been misclassified as lymphangiosarcomas. Nevertheless, canine HSA is typically subclassified based on the primary site: visceral HSA (splenic and hepatic HSA), cardiac HSA, and cutaneous HSA (13). The prognosis is typically grave in splenic, hepatic, and cardiac HSA, with median survival times ranging from 19 to 179 days (23–30). On the other hand, cutaneous HSA has a relatively favorable prognosis, with median survival times ranging from 307 to 1189 days (31, 32).

## Epidemiology

Human AS is a very rare cancer, accounting for less than 0.01% of all adult malignancies (14, 33). AS is more likely to occur on the skin of white, elderly individuals, but there are no significant differences in distribution between sexes (33). While AS can arise from any soft tissue or organ with vascular tissues, it most commonly affects the skin of the head, neck, scalp, breast, and extremities. Visceral forms of AS, occurring in the liver, right atrium of the heart, and spleen, are less frequent.

Dogs have a significantly higher incidence of HSA compared to humans, with an estimated 25 to 100 times higher incidence rate (34). HSA accounts for 5% of all non-cutaneous malignant neoplasms in dogs (13, 34) and approximately 50% of all splenic tumors (13, 35). Similar to humans, HSA predominantly affects older animals, although there seems to be a slight male predisposition in dogs (36, 37). Golden Retrievers and German Shepherds are high-risk breeds, and HSA is the leading cause of cancer-associated death in Golden Retrievers (28, 38). Like in humans, HSA can arise from any soft tissue with vasculature, but it most commonly affects the spleen, right atrium of the heart, liver, and skin or subcutaneous tissue (13). The different anatomical distribution observed in human AS and canine HSA is one of the characteristic differences, although the exact underlying cause is unknown.

## Etiology

Multiple potential risk factors have been identified in human AS, including chronic lymphedema, radiotherapy, UV radiation, BRCA mutation, familial syndromes, chemical exposure, foreign bodies, and immunosuppression. Radiotherapy and chronic lymphedema are well-known risk factors for AS (39, 40). Breast cancer patients who receive adjuvant radiotherapy are predisposed to developing chronic lymphedema and subsequent breast AS as unintended side effects of treatment. This condition is known as Stewart-Treves syndrome (39). The highest incidence of AS in breast cancer patients occurs 5-10 years after adjuvant radiotherapy (40). The risk of AS further increases in patients with mutations in the *BRCA1* (185delAG) and *BRCA2* (854delC) genes (41), which are crucial for DNA repair. Milroy’s disease and chronic filariasis also cause chronic lymphedema and are associated with the development of AS (15). AS is associated with various familial syndromes such as neurofibromatosis, Maffucci syndrome, Li-Fraumeni syndrome (*TP53* mutations), and Klippel-Trenaunay syndrome (*PIK3CA* mutations) (15), which is consistent with the observed profiles of recurrent mutations in AS. Chemical exposure is another risk factor for AS development, including exposure to vinyl chloride (42), thorium dioxide (43), arsenic, radium, and anabolic steroids (44). Among these, vinyl chloride and thorium dioxide predominantly induce hepatic AS (42, 43). Foreign bodies can also cause AS, such as surgical gauzes (45), vascular prostheses (46), orthopedic prostheses (47), and gouty tophus (48). The causal association between immunosuppression and AS tumorigenesis remains unclear; however, AS has been observed in immunosuppressed patients following renal transplantation, and some epidemiological studies suggest a potential association between AS and AIDS (49).

Not many risk factors have been reported for canine HSA so far; however, the identified risk factors include dog breeds such as German Shepherds or Golden Retrievers (50) and UV radiation (51, 52). Dog breeds such as German Shepherds or Golden Retrievers are considered strong risk factors, possibly due to genetic imbalances resulting from intense inbreeding and selection. A genome-wide association study reported several loci significantly associated with the risk of HSA in Golden Retrievers (50). Like human AS, UV radiation has also been associated with cutaneous HSA (51, 52), which exhibits a high mutation rate and a strong UV mutational signature (53).

## Pathology

Both human AS and canine HSA are heterogeneous tumors with significant intra- and intertumoral differences. The hallmark of AS is the proliferation of pleomorphic endothelial cells showing rounded, polygonal, fusiform, or epithelioid morphology without a clear border to normal tissue. Well-differentiated AS may contain abnormal endothelial cells forming vascular sinusoids continuous with normal vascular channels; however, aggressive and poorly differentiated AS tends to lose such architecture and has an epithelioid morphology with a high mitotic rate and areas of hemorrhage and necrosis (15, 54, 55), which grants AS tissue prominent complexity and heterogeneity. AS typically expresses endothelial markers including von Willebrand factor, CD34, CD31, and VEGF. Among these, von Willebrand factor (15) and CD31 are the most commonly used markers for distinguishing AS from other undifferentiated neoplasms (56). However, progressive AS may lose these expressions and gain expression of cytokeratins, which can cause confusion with undifferentiated epithelial malignancies (56).

Canine HSA exhibits similar histological characteristics to human AS. The histologic features include the proliferation of immature, pleomorphic endothelial cells with expression of von Willebrand factor, CD31, claudin-5, CD117, and VEGF (57–61). Similarly, von Willebrand factor and CD31 are the most commonly used markers for distinguishing HSA from other malignancies. An intriguing hypothesis regarding the origin of canine HSA has recently been proposed. Canine HSA has been considered to originate from vascular endothelium based on histopathological findings. However, recent studies have revealed that these malignant cells may originate from pluripotent bone marrow progenitors at the stage of hemangioblasts to angioblasts differentiating into endothelial cells (34, 62, 63). This hypothesis has been reported only in canine HSA; however, given the similarities between human AS and canine HSA, it may also be relevant to the origins of human AS.

## Molecular abnormalities

**Figure 1:**
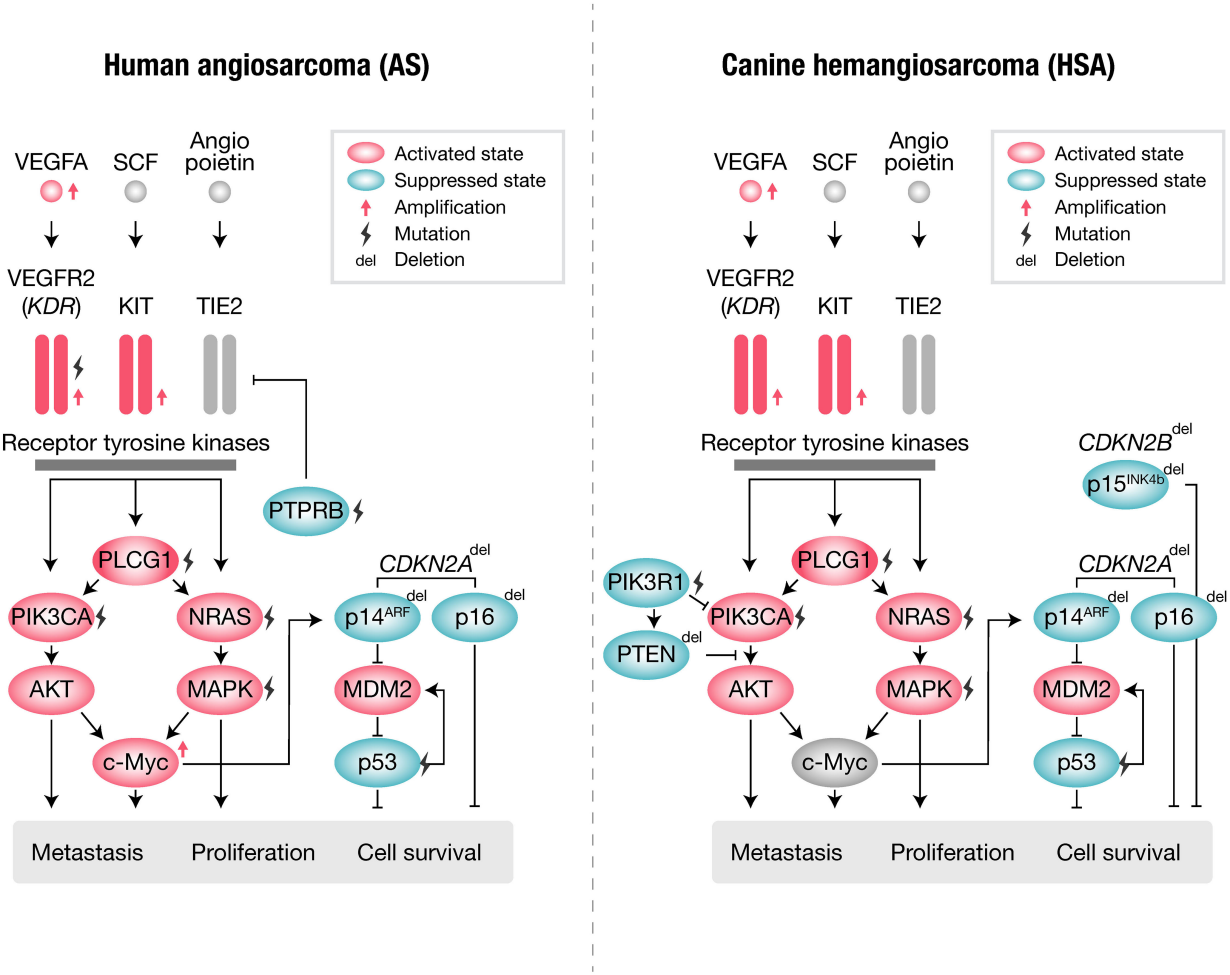
Summary of gene abnormalities in human angiosarcoma (AS) and canine hemangiosarcoma (HSA).

Human AS and canine HSA share many similar genetic abnormalities with minor differences (**Figure 1**, **Table 1**). Human AS reportedly has many types of molecular abnormalities; however, common driver pathogenic mutations or copy number aberrations shared in all reported cases have not been identified, likely due to the high heterogeneity in primary locations or etiologic factors of AS. Recurrent mutations in human AS include *KDR* (VEGFR2) (64), *TP53* (67, 73), and *PIK3CA* (64). Other genetic abnormalities of AS include mutations of *KRAS* (69, 74), MAPK (65), *PTPRB*, and *PLCG* (70), as well as amplifications of *KDR* (65, 75), *VEGFA* (72, 73), *MYC* (65, 75), *KIT* (64), and deletions of *CDKN2A* (65, 75). Among these, *PTPRB* and *PLCG* mutations and *MYC* amplification are most frequently observed in secondary AS, such as radiation-induced AS. Other factors are also reported in association with AS pathogenesis: overexpression of *WT1* (Wilms Tumor 1) (76), *LGALS3* (Galectin-3) (77), *ETS1*, metalloproteinases (MMP1, MMP3, and uPA) (78, 79), and *FSCN* (the actin-bundling motility protein) (72). AS has a *KIT* expression (80–82) and amplification (64), whereas no activating mutations in exons 11 (juxtamembrane domain) or 17 (kinase domain) of *KIT* have been observed so far.

**Table 1:** Gene abnormalities shared between human angiosarcoma (AS) and canine hemangiosarcoma (HSA).

	Human AS		Canine HSA	
	Mutation	Amplification	Mutation	Amplification
KDR (VEGFR2)	12/47 (26%) Painter et al. (64)	4/34 Murali et al. (65)	–	14/47 (22%) Megquier et al. (66)
TP53	14/47 (30%) Painter et al. (64) 7/13 (53.8%) Naka et al. (67)	–	33/50 (66%) Wang et al. (68) 14/15 (93.33%) Wong et al. (53)	–
PIK3CA	10/47 (21%) Painter et al. (64)	–	23/50 (46%) Wang et al. (68) 14/47 (29.8%) Megquier et al. (66)	–
RAS	NRAS 3/47 (6%) Painter et al. (64) KRAS 8/15 (53.3%) Weihrauch et al. (69)	–	NRAS 12/50 (24%) Wang et al. (68)	–
PLCG	8/47 (17%) Painter et al. (64) 3/34 (8.8%) Behjati et al. (70)	–	2/50 (4%) Wang et al. (68) 1/20 (5%) Wang et al. (71)	–
VEGFA	–	20/20 (100%) Dim et al. (72)	–	9/47 (19%) Megquier et al. (66)
KIT	–	4/47 (9%) Painter et al. (64)	–	8/47 (17%) Megquier et al. (66)
CDKN2A (p14ARF, p16)	Deletion 9/34 (26%) Murali et al. (65)	–	Deletion 10/47 (22%) Megquier et al. (66)	–

Angiogenic pathways have long been recognized for their pivotal roles in the context of Angiosarcoma (AS). Specifically, the central components of these angiogenic pathways, namely *KDR* (VEGFR2) and *VEGFA*, often exhibit mutations and amplifications, as documented in multiple studies (64, 72, 73). KDR serves as the principal receptor tyrosine kinase responsible for mediating VEGFA-induced proangiogenic signaling. As such, it was initially anticipated that the loss of KDR signaling might suppress the proliferation and metastasis of AS, correlating with a more favorable prognosis. Intriguingly, however, the results proved contrary, revealing that the loss of KDR is associated with a less favorable prognosis (83). These findings suggest that KDR may not be a contributing factor to the tumorigenesis of AS but rather a potential regulator of endothelial cell differentiation. This may be relevant to the disappointing results of clinical trials for bevacizumab, an antibody targeting VEGF. In a phase II study, only 2 out of 23 patients achieved partial response, and 11 had stable disease (84), and even when combined with Paclitaxel, the response rate was only 28% compared to 45.8% in the combination or monotherapy with Paclitaxel (85).

RAS and its downstream pathways appear to play important roles in AS. The mutation of *NRAS* and *KRAS* has been observed in AS (64, 69, 74). Furthermore, a functional study revealed that the introduction of continuously activated *HRAS* into murine endothelial cells produces poorly differentiated AS *in vivo* (86), indicating essential roles of the RAS pathway in the tumorigenesis of AS. Among the downstream pathways of RAS, the activation of the PI3K pathway has been frequently documented in AS patients. Of note, several studies have suggested that PI3K signaling is more important than the MAPK signaling cascade in AS (86, 87). Although activation of PI3K pathways has been frequently noted, the mutation of *PIK3CA* itself had not been reported in AS studies. However, a recent international cooperative project (Angiosarcoma Project) revealed that the *PIK3CA* activating mutation was one of the most frequently mutated genes in human AS (64). These reports suggest a strong contribution of PI3K pathways to the tumorigenesis of AS.

Canine HSA has been reported to bear many shared genetic abnormalities with human AS. The most commonly observed recurrent mutations in canine HSA include *TP53* (66), *PIK3CA* (activating) (66, 71), and *PIK3R1* (66), *PTEN* (inactivating) (71), *NRAS* (68), and *PLCG1* (66, 68). Additionally, canine HSA frequently has other genetic abnormalities, including the deletion of *CDKN2A/B* (66) and *PTEN* (88) and the amplification of *KDR*, *VEGFA*, and *KIT* (66). However, unlike in human AS, the amplification of *MYC* is not frequently observed in canine HSA (89). This is likely due to secondary HSA not being prevalent in dogs.

Many components of the angiogenic pathways are also altered in canine HSA. Consistent with the profiles of human AS, canine HSA frequently exhibits activation of the VEGFA-KDR pathway (66). Similarities have also been observed in the clinical response to inhibitors of angiogenic pathways. Despite the predominant activation of the VEGFA-KDR pathway, a small molecule inhibitor targeting canine KDR, toceranib, failed to induce sufficient clinical responses in canine HSA (90). The results from veterinary clinical trials strongly suggest fundamental similarities in the response to treatment between human AS and canine HSA.

Canine HSA also shares gene abnormalities in the RAS and PI3K pathways, as observed in human AS (68, 71). Intriguingly, activating mutations in *PIK3CA* were first identified in canine HSA by veterinary researchers (71). Initially, these activating mutations in *PIK3CA* were considered to be specific to canines. However, a recent large-scale analysis (Angiosarcoma Project) revealed that human AS also frequently has activating mutations in *PIK3CA* (64). This exemplifies that canine HSA has highly similar molecular characteristics to human AS, indicating the strong potential of canine HSA as a model with similar molecular and clinical characteristics.

## Treatment

Currently, there are no effective therapeutic options for both human AS and canine HSA. Human AS has seldom been the primary focus of clinical trials due to its uncommon occurrence; instead, it has frequently been included in prospective clinical trials as part of the sarcoma in general without specialized treatment. To date, there have been no prospective clinical trials for AS that demonstrated a prominent survival benefit of systemic therapy in either the neoadjuvant or adjuvant setting. In line with this, many of the discussions regarding therapeutic options for AS are based on limited evidence reported from retrospective studies, albeit with a few prospective studies.

The primary choice of treatment for human AS with localized lesions remains radical surgery with complete resection. However, due to the invasive nature of AS, achieving complete resection with clear margins is often challenging (17, 91–93). Given the circumstances, neoadjuvant chemotherapy may be performed using gemcitabine, docetaxel, doxorubicin, ifosfamide, and paclitaxel; however, no survival benefit has been reported with the addition of neoadjuvant chemotherapy (94). Concurrent therapy with paclitaxel and radiation has been explored for localized cutaneous AS, and a prospective study has shown improved survival with a 2-year overall survival (OS) of 94.1% compared to 71.6% in the control group (18). In the view of treating metastatic disease, several cytotoxic drugs and regimens are used for this purpose. One of the most commonly used agents is taxane-based regimens involving paclitaxel. A Phase II single arm clinical trial demonstrated that paclitaxel has particular activity in AS and is now often used in the first or second-line setting (18). A prospective study showed that the response rate for this treatment was approximately 19%, with a median progression-free survival (PFS) of 4 months and OS of 8 months (19). Anthracycline-based regimens using doxorubicin are also often utilized, with a response rate of approximately 25%, and a median PFS of 4.9 months and OS of 9.9 months (prospective study) (95).

Canine visceral HSA is usually treated with surgery and adjuvant chemotherapy, and most of the prognostic information in the literature is from retrospective analysis. For splenic HSA, total splenectomy and adjuvant chemotherapy with a single agent or combination protocols involving doxorubicin are recommended (13). However, the therapeutic efficacy is limited, and significant improvement in survival time is rarely achieved. Splenectomy alone has shown median survival times ranging from 19 to 86 days (23–27), with a 2-month survival rate of 31% and a 1-year survival rate of 7% (96). Surgery and adjuvant chemotherapy have resulted in median survival times of 141–179 days, with less than 10% of dogs surviving beyond one year (28–30). Cardiac HSA treated with surgical excision and adjuvant doxorubicin-based chemotherapy showed a similar response to splenic HSA, with median survival times of 183–189 days (97, 98). For cutaneous HSA, surgery with or without adjuvant chemotherapy is generally performed (13). Wider margins are recommended for surgery, although it may be difficult depending on the tumor location, similar to human AS (13). Cutaneous HSA has median survival times of 307 days (with invasion into the surrounding tissue) or greater than 2 years (without evidence of invasion), which is a much better prognosis compared to visceral HSA (31). Cutaneous HSA treated with surgery and adjuvant doxorubicin has shown median survival times as long as 1189 days (32). Only a few studies have reported the efficacy of radiation therapy for canine HSA, and none have reported a significant improvement in overall survival (99).

## Other treatment options and potential treatment under investigation

For the treatment of human AS, several targeted agents have been explored as alternative options. Agents targeting the VEGF-VEGFR angiogenic pathway have been assessed for sarcoma, including bevacizumab and pazopanib. Bevacizumab, a monoclonal antibody targeting VEGFA (84, 100, 101), produced modest results with only 2 out of 23 patients showing a partial response and 11 out of 23 patients with stable disease in a Phase II clinical trial (84). The results were disappointing considering the well-known alteration of the VEGFA-VEGFR pathway in AS. Pazopanib, a tyrosine kinase inhibitor, also yielded modest results with a median progression-free survival (PFS) of 3 months and no significant responses in a retrospective study (102). Immune checkpoint inhibitors (ICIs) have recently emerged as another option for AS. The anti-PD1 (programmed death 1) checkpoint inhibitor pembrolizumab was approved for tumors with a high tumor mutation burden regardless of histology (103). Pembrolizumab showed an exceptional and durable response to 2 out of 3 metastatic AS cases that were refractory to standard therapies (64). Although further studies are required to confirm their efficacy, these reports suggest the promising potential of ICIs for AS treatment.

For canine HSA, multiple agents have been explored as alternative options, including a tyrosine kinase inhibitor targeting KDR (toceranib) (90), a taxane-based agent (Paccal-Vet) (104), immune checkpoint inhibitors (ICIs) (105, 106), other forms of anthracycline (epirubicin) (107), pegylated liposome-encapsulated doxorubicin (108), COX-2 inhibitors (109), and thalidomide (110). Toceranib, a tyrosine kinase inhibitor targeting KDR (90), has been explored for the treatment of canine HSA; however, the results were disappointing: a prospective study showed that the use of toceranib following doxorubicin-based chemotherapy did not improve either disease-free interval or OS in stage I or II canine HSA (a median disease-free interval, 161 days; a median survival time, 172 days) (90). Paccal Vet, a water-soluble, micellar formulation of paclitaxel, has been investigated for treating canine HSA. Paclitaxel has not been used in dogs due to high rates of hypersensitivity reactions when given intravenously (111, 112); however, Paccal Vet is designed not to induce such hypersensitivity, thus it is expected to be useful for the treatment of canine HSA (104). Although ICIs had not been commercially available for dogs, Gilvetmab, the first anti-canine PD-1 antibody, has been approved in October 2023 (Merck Animal Health USA). Currently, Gilvetmab is approved only for 2 tumor types (mast cell tumor and malignant melanoma), and its clinical efficacy for canine HSA is still unclear. Although further studies are required, ICIs have promising potential as a therapeutic modality for canine HSA as in human AS. Several other forms of conventional immunotherapy are documented, including an HSA vaccine (113), liposome-encapsulated muramyl tripeptide phosphatidylethanolamine (LMTP-PE) (114), and polysaccharopeptide (115); however, their efficacy may be limited.

## Issues in drug discovery research for human AS

Major issues associated with drug discovery research for human AS are caused by its rarity and can be subdivided into scientific or investment issues. The scientific issues include (1) lack of comprehensive information regarding treatment and biological characteristics and (2) the lack of appropriate research models.

The majority of information regarding therapeutic responses and biological characteristics of AS is based on the results from case series, which may possess potential bias in selection, and the dataset may be incomplete or a mixture of the results from different treatment approaches (12). Randomized trials are lacking, and there are limited prospective studies (12). Nonetheless, recent initiatives for international collaboration, such as The Angiosarcoma Project (64) and the International Rare Diseases Research Consortium (IRDiRC), hold promise for gradually addressing these challenges.

Cell lines and conventional xenograft mouse models useful for AS research are also lacking. The establishment of AS cell lines is extremely difficult. As a result, well-characterized and commonly used cell lines are limited to only a few, including ISO-HAS (116) and ASM (117). Similarly, the availability of cell-derived xenograft models (CDX) and patient-derived xenograft models (PDX) is limited, although a recent study reported a rare successful example (118).

The low incentives for pharmaceutical companies to invest in new drugs for AS are another challenge (119). Drug development specifically for AS carries a high risk of failing to recoup the invested funds and is thus rarely pursued. Additionally, few clinical trials evaluating new compounds for other major cancers include patients with rare cancers such as AS. This is because the inclusion of such patients increases the risk of experiencing unexpected adverse events, which could critically halt the entire development process. Furthermore, the additional costs related to managing compound supply, developing companion diagnostics, and applying for regulatory approvals are not easily justified as investments for pharmaceutical companies. Given these circumstances, current drug discovery research for AS mainly focuses on drug repositioning studies that can be conducted with relatively low-risk investments. However, relying solely on these drug repositioning studies remains challenging to achieve the development of truly effective therapeutic drugs for AS.

The inefficient collection of patients for clinical trials or clinical samples has been a factor that hinders drug discovery research for AS. However, this is getting less problematic due to recently improved cooperation between specialized hospitals or institutions for the registration and enrollment of clinical trials (64).

## Advantages and disadvantages of canine HSA as a drug discovery model

**Figure 2:**
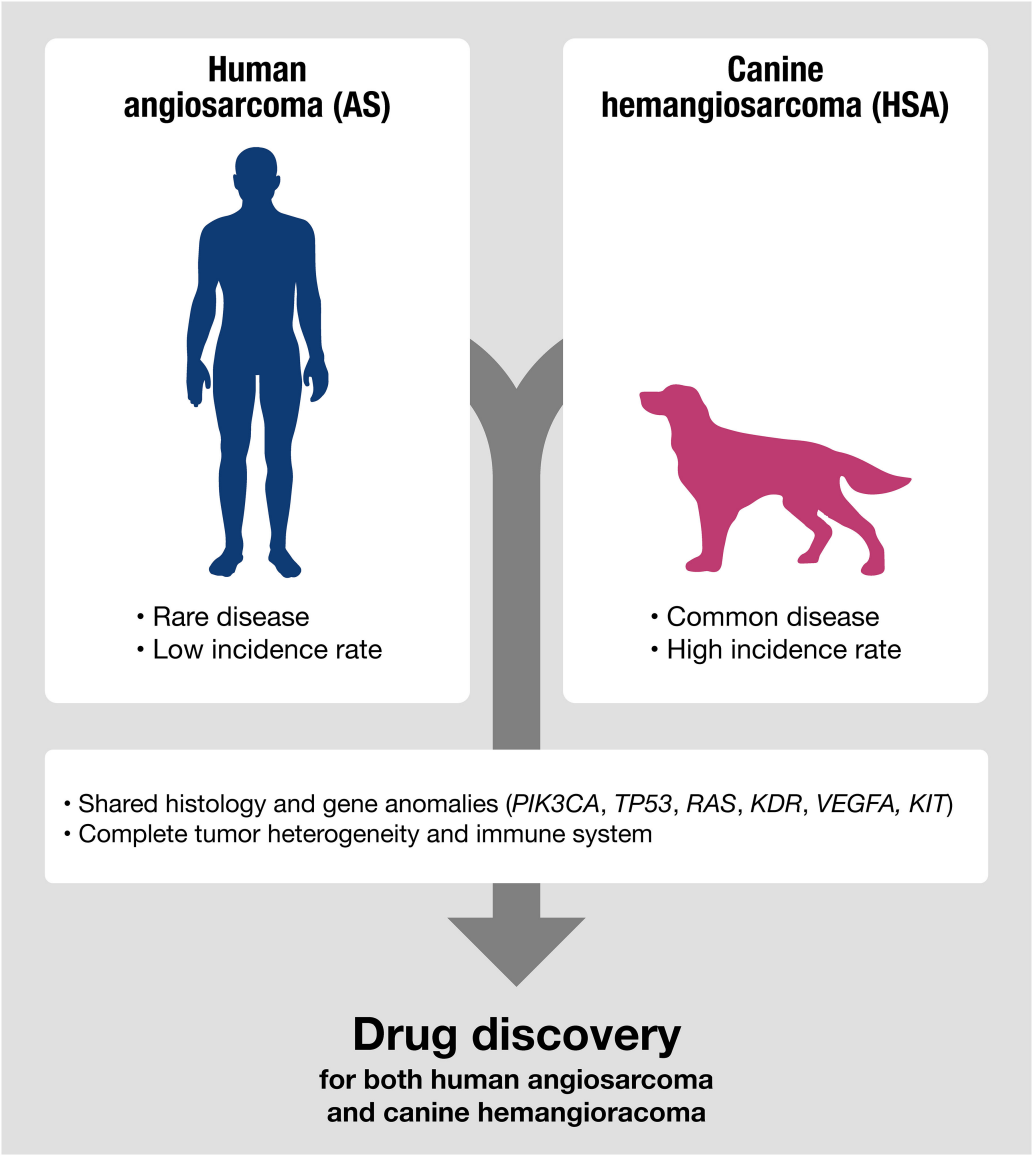
Canine HSA shares molecular signatures with human AS, making it a potentially useful model for predicting therapeutic responses in human AS, especially for evaluating the efficacy of drugs targeting shared genetic anomalies between humans and dogs.

Canine HSA has advantages as a non-conventional model for drug discovery (**Figure 2**). Despite canine HSA having an exceptionally higher incidence rate than its human analog, it retains significant similarities with human AS, including multistep tumorigenesis that occurs over several years, histological and molecular characteristics, a complete immune system, intra/intertumoral heterogeneity, and living environment. Furthermore, many HSA cell lines have been established and are available for basic research (63, 113, 120–125). The value of the dog model is further highlighted given the advantages in the investigation of immunotherapy. Since the recent broad applications of ICIs, understanding clinical responses to immunotherapy in the heterogeneous tumor microenvironment seen in human cancers is gaining more importance. However, conventional rodent models hardly serve as appropriate models for this purpose due to their non-spontaneous and artificial nature of tumors with dysfunctional immune systems. In contrast, canine HSA is a naturally occurring tumor, which has genetic heterogeneity and an intact immune system that could closely recapitulate the complexity of human cancers. The recent development of a canine version of ICI (Gilvetmab) could further facilitate the investigation of clinical responses to immunotherapy in the setting of genetic and immunologic heterogeneity of tumors. These characteristics and the research environment are useful for analyzing therapeutic responses and the complexities of drug resistance, metastasis, and tumor-host immune interactions in AS patients.

On the other hand, the strategy of using canine HSA as a drug discovery model also has certain disadvantages to consider. Conducting drug discovery research using canine HSA in a clinical setting could be more expensive compared to conventional preclinical studies using laboratory animals such as mice and rats. Dogs have a larger body size than laboratory animals, which requires a higher amount of drugs for testing. Additionally, to acquire high-quality clinical data to evaluate the response to drugs, frequent medical checks during administration may be necessary. These medical checks typically include a set of assessments such as complete blood cell count, blood biochemistry, urinalysis, ultrasound, X-ray, computed tomography (CT), and magnetic resonance imaging (MRI), which significantly increases the total costs. Moreover, systemic anesthesia is typically required for dogs undergoing CT and MRI, adding extra costs and medical risks to the process of performing these evaluations. Sometimes systemic anesthesia could result in fatal situations, particularly for elderly dogs with cancer, making it challenging to perform these evaluations multiple times from an animal welfare perspective. Furthermore, from a regulatory perspective, the role and purpose of using spontaneous dog tumor models are not clearly defined in the status quo. Therefore, the data collected from canine trials may be considered as supplemental information, which may not have significant impacts on drug approval. Additionally, drug repositioning studies can be directly initiated with humans, reducing the relative necessity of canine models for such purposes. Moreover, although many similarities have been reported between human AS and canine HSA, these diseases are not entirely identical. Therefore, for each study, performing appropriate basic research is necessary to validate that the target mechanism of action is actually conserved between human AS and canine HSA.

## Potentials as a model for drug efficacy evaluation

As discussed above, canine HSA is a potentially useful model for evaluating drug efficacy; however, more research is needed to fully utilize its potential. To facilitate the effective use of this model in drug discovery, several key factors should be considered from the current regulatory perspective. The most critical factor to consider is the scientific validity of the model, given that there are currently no established regulatory guidelines for utilizing canine HSA in evaluating drug efficacy (126). Scientific validity is typically determined by whether the model appropriately reflects the molecular mechanisms, physiological conditions, and microenvironment of the target tumors in humans (126). Therefore, conducting basic research to uncover the more detailed characteristics of HSA, as well as establishing guidelines for model evaluation by academic societies, can be an effective strategy to promote the use of canine HSA as a valuable tool in drug discovery. Canine HSA, in this role, holds the potential to help mitigate the risks associated with drug development for human AS. This can be accomplished by early identification of agents with promising activity and safety, effectively distinguishing them from those likely to fail in the drug development journey. Such an approach represents a promising avenue for advancing AS drug development and research progress.

Canine HSA may be useful for basic research aimed at identifying novel mechanisms or therapeutic targets for the development of new drugs, given that it appears to share fundamental similarities with human AS in terms of biological characteristics and therapeutic responses to drugs. Nonetheless, the investigation of new drugs for AS may be challenging due to non-scientific reasons, including low incentives for investing in drug discovery for rare cancers, as mentioned in a previous section. However, a recent approach that targets a limited disease population and recovers the investment through increased drug prices (low population/high margin) may help overcome this situation in drug discovery for rare cancers (119). Several new drugs for rare diseases have been successfully developed using this approach, such as Zolgensma for spinal muscular atrophy (affecting 1 out of 10,000 people) (127) and Tofersen (128) for SOD1-mutated amyotrophic lateral sclerosis (affecting 3 out of 1,000,000 people). Utilizing such an approach may help overcome the current situation filled with difficulties, and the results obtained from basic research using canine HSA still have the potential to pave the way for future new drug development for AS. Additionally, canine HSA may also be a useful research model to explore appropriate doses and intervals in exploratory studies involving the repositioning of drugs that are approved for other cancers. Also, the heterogeneous nature of canine HSA, which includes the complexity of the tumor microenvironment with a complete immune system, may help collect crucial information for understanding factors essential for drug resistance and recurrence.

## Potentials as a model for toxicity evaluation

Canine HSA may not be an effective model for toxicity evaluation in drug development as it does not meet the standards of Good Laboratory Practice (GLP) studies. Companion dogs with HSA lack sufficient background or reference data, making it difficult to interpret the results and reducing the reliability of the data compared to toxicity studies conducted under GLP conditions. The nature of this model makes it challenging to determine whether the observed toxicity is caused by disease exacerbation, drug effects, or individual differences within the model. This critical disadvantage hinders the scientific investigation of the underlying causes of toxicity observed in the model. The use of non-conventional animal models may be considered only when general toxicity evaluations are deemed inappropriate. One example is biopharmaceuticals that do not exhibit pharmacological effects in conventional animal models. In such cases, non-conventional animal models can be justified by examining species differences in terms of pharmacological effects, target molecule distribution and expression, and tumor behavior, with reference to ICH guideline S6 (129). However, it is unrealistic to check all these aspects in companion animals; therefore, transgenic animals are commonly used as substitutes for this purpose. The results of efficacy studies described in the previous section may be included as supplemental information for safety evaluations. However, they are typically considered as supplemental reference data with less impact on drug approvals. Therefore, the companion animal model cannot provide the same quality of toxicity data, limiting its use in toxicological studies.

## Conclusion

The development of new drug discovery for AS has remained elusive, and the challenges outlined in this review persist. However, it is crucial that these limitations do not deter the commitment of our research community to advancing therapeutic options for patients with AS. As illustrated in this review, canine HSA exhibits significant molecular similarities to human AS, making it a valuable model for predicting therapeutic responses in human AS, especially when evaluating the efficacy of drugs targeting shared genetic anomalies between humans and dogs. This review represents an initial step toward the development of novel drugs for AS and HSA. Nevertheless, further foundational research is imperative to enhance the utilization of canine HSA as a model for drug discovery.

## Author contributions

KH, NA, MH, and AK wrote the first draft of the manuscript. All authors contributed to the article and approved the submitted version.

## References

[1] Axelsson E Ratnakumar A Arendt ML Maqbool K Webster MT Perloski M. The genomic signature of dog domestication reveals adaptation to a starch-rich diet. Nature (2013) 495(7441):360–4. doi: 10.1038/nature11837 23354050

[2] Davis SJ Valla FR. Evidence for domestication of the dog 12,000 years ago in the Natufian of Israel. Nature (1978) 276(5688):608–10. doi: 10.1038/276608a0

[3] Pang JF Kluetsch C Zou XJ Zhang AB Luo LY Angleby H. Mtdna data indicate a single origin for dogs south of Yangtze River, less than 16,300 years ago, from numerous wolves. Mol Biol Evol (2009) 26(12):2849–64. doi: 10.1093/molbev/msp195 PMC2775109 19723671

[4] Skoglund P Gotherstrom A Jakobsson M. Estimation of population divergence times from non-overlapping genomic sequences: examples from dogs and wolves. Mol Biol Evol (2011) 28(4):1505–17. doi: 10.1093/molbev/msq342 21177316

[5] Wang GD Zhai W Yang HC Fan RX Cao X Zhong L. The genomics of selection in dogs and the parallel evolution between dogs and humans. Nat Commun (2013) 4:1860. doi: 10.1038/ncomms2814 23673645

[6] Lindblad-Toh K Wade CM Mikkelsen TS Karlsson EK Jaffe DB Kamal M. Genome sequence, comparative analysis and haplotype structure of the domestic dog. Nature (2005) 438(7069):803–19. doi: 10.1038/nature04338 16341006

[7] Sutter NB Eberle MA Parker HG Pullar BJ Kirkness EF Kruglyak L. Extensive and breed-specific linkage disequilibrium in Canis familiaris. Genome Res (2004) 14(12):2388–96. doi: 10.1101/gr.3147604 PMC534662 15545498

[8] Wilbe M Jokinen P Truve K Seppala EH Karlsson EK Biagi T. Genome-wide association mapping identifies multiple loci for a canine Sle-related disease complex. Nat Genet (2010) 42(3):250–4. doi: 10.1038/ng.525 20101241

[9] Olsson M Meadows JR Truve K Rosengren Pielberg G Puppo F Mauceli E. A novel unstable duplication upstream of Has2 predisposes to a breed-defining skin phenotype and a periodic fever syndrome in Chinese Shar-Pei dogs. PloS Genet (2011) 7(3):e1001332. doi: 10.1371/journal.pgen.1001332 21437276 PMC3060080

[10] Seppala EH Jokinen TS Fukata M Fukata Y Webster MT Karlsson EK. Lgi2 truncation causes a remitting focal epilepsy in dogs. PloS Genet (2011) 7(7):e1002194. doi: 10.1371/journal.pgen.1002194 21829378 PMC3145619

[11] Downs LM Wallin-Hakansson B Boursnell M Marklund S Hedhammar A Truve K. A frameshift mutation in golden retriever dogs with progressive retinal atrophy endorses Slc4a3 as a candidate gene for human retinal degenerations. PloS One (2011) 6(6):e21452. doi: 10.1371/journal.pone.0021452 21738669 PMC3124514

[12] Young RJ Brown NJ Reed MW Hughes D Woll PJ. Angiosarcoma. Lancet Oncol (2010) 11(10):983–91. doi: 10.1016/S1470-2045(10)70023-1 20537949

[13] Vail DM Thamm DH Liptak JM. Withrow and Macewen's Small Animal Clinical Oncology. Oxford, UK: Elsevier Health Sciences (2019).

[14] Lahat G Dhuka AR Hallevi H Xiao L Zou C Smith KD. Angiosarcoma: clinical and molecular insights. Ann Surg (2010) 251(6):1098–106. doi: 10.1097/SLA.0b013e3181dbb75a 20485141

[15] Weiss SW Goldblum JR Folpe AL. Enzinger and Weiss's Soft Tissue Tumors. Oxford, UK: Elsevier Health Sciences (2007).

[16] Schlemmer M Reichardt P Verweij J Hartmann JT Judson I Thyss A. Paclitaxel in patients with advanced angiosarcomas of soft tissue: a retrospective study of the EORTC soft tissue and bone sarcoma group. Eur J Cancer (2008) 44(16):2433–6. doi: 10.1016/j.ejca.2008.07.037 18771914

[17] Fury MG Antonescu CR Van Zee KJ Brennan MF Maki RG. A 14-year retrospective review of angiosarcoma: clinical characteristics, prognostic factors, and treatment outcomes with surgery and chemotherapy. Cancer J (2005) 11(3):241–7. doi: 10.1097/00130404-200505000-00011 16053668

[18] Florou V Wilky BA. Current management of angiosarcoma: recent advances and lessons from the past. Curr Treat Options Oncol (2021) 22(7):61. doi: 10.1007/s11864-021-00858-9 34097172

[19] Penel N Bui BN Bay JO Cupissol D Ray-Coquard I Piperno-Neumann S. Phase II trial of weekly paclitaxel for unresectable angiosarcoma: the angiotax study. J Clin Oncol (2008) 26(32):5269–74. doi: 10.1200/JCO.2008.17.3146 18809609

[20] Kelly WR Wilkinson GT Allen PW. Canine angiosarcoma (Lymphangiosarcoma). Vet Pathol (1981) 18(2):224–7. doi: 10.1177/030098588101800210 7467082

[21] Curran KM Halsey CH Worley DR. Lymphangiosarcoma in 12 dogs: A case series (1998-2013). Vet Comp Oncol (2016) 14(2):181–90. doi: 10.1111/vco.12087 24612140

[22] Halsey CH Worley DR Curran K Charles JB Ehrhart EJ. The use of novel lymphatic endothelial cell-specific immunohistochemical markers to differentiate Cutaneous angiosarcomas in dogs. Vet Comp Oncol (2016) 14(3):236–44. doi: 10.1111/vco.12088 24593773

[23] Prymak C McKee LJ Goldschmidt MH Glickman LT. Epidemiologic, clinical, pathologic, and prognostic characteristics of splenic hemangiosarcoma and splenic hematoma in dogs: 217 cases (1985). J Am Vet Med Assoc (1988) 193(6):706–12. 3192450

[24] Wood CA Moore AS Gliatto JM Ablin LA Berg RJ Rand WM. Prognosis for dogs with stage I or II splenic hemangiosarcoma treated by splenectomy alone: 32 cases (1991-1993). J Am Anim Hosp Assoc (1998) 34(5):417–21. doi: 10.5326/15473317-34-5-417 9728473

[25] Goritz M Muller K Krastel D Staudacher G Schmidt P Kuhn M. Canine splenic haemangiosarcoma: influence of metastases, chemotherapy and growth pattern on post-splenectomy survival and expression of angiogenic factors. J Comp Pathol (2013) 149(1):30–9. doi: 10.1016/j.jcpa.2012.11.234 23276383

[26] Batschinski K Nobre A Vargas-Mendez E Tedardi MV Cirillo J Cestari G. Canine visceral hemangiosarcoma treated with surgery alone or surgery and doxorubicin: 37 cases (2005-2014). Can Vet J (2018) 59(9):967–72. PMC6091137 30197439

[27] Wendelburg KM Price LL Burgess KE Lyons JA Lew FH Berg J. Survival time of dogs with splenic hemangiosarcoma treated by splenectomy with or without adjuvant chemotherapy: 208 cases (2001–2012). J Am Vet Med (2015) 247(4):393–403. doi: 10.2460/javma.247.4.393 26225611

[28] Brown NO Patnaik AK MacEwen EG. Canine hemangiosarcoma: retrospective analysis of 104 cases. J Am Vet Med Assoc (1985) 186(1):56–8. 4038395

[29] Hammer AS Couto CG Filppi J Getzy D Shank K. Efficacy and toxicity of ac chemotherapy (Vincristine, doxorubicin, and cyclophosphamide) in dogs with hemangiosarcoma. J Vet Intern Med (1991) 5(3):160–6. doi: 10.1111/j.1939-1676.1991.tb00943.x 1920253

[30] Lana S U'Ren L Plaza S Elmslie R Gustafson D Morley P. Continuous low-dose oral chemotherapy for adjuvant therapy of splenic hemangiosarcoma in dogs. J Vet Intern Med (2007) 21(4):764–9. doi: 10.1892/0891-6640(2007)21[764:clocfa]2.0.co;2 17708397

[31] Ward H Fox LE Calderwood-Mays MB Hammer AS Couto CG. Cutaneous hemangiosarcoma in 25 dogs: A retrospective study. J Vet Intern Med (1994) 8(5):345–8. doi: 10.1111/j.1939-1676.1994.tb03248.x 7837111

[32] Bulakowski EJ Philibert JC Siegel S Clifford CA Risbon R Zivin K. Evaluation of outcome associated with subcutaneous and intramuscular hemangiosarcoma treated with adjuvant doxorubicin in dogs: 21 cases (2001-2006). J Am Vet Med Assoc (2008) 233(1):122–8. doi: 10.2460/javma.233.1.122 18593321

[33] Rouhani P Fletcher CD Devesa SS Toro JR. Cutaneous soft tissue sarcoma incidence patterns in the U.S. : an analysis of 12,114 cases. Cancer (2008) 113(3):616–27. doi: 10.1002/cncr.23571 18618615

[34] Kim JH Graef AJ Dickerson EB Modiano JF. Pathobiology of hemangiosarcoma in dogs: research advances and future perspectives. Vet Sci (2015) 2(4):388–405. doi: 10.3390/vetsci2040388 29061949 PMC5644642

[35] Spangler WL Culbertson MR. Prevalence, type, and importance of splenic diseases in dogs: 1,480 cases (1985-1989). J Am Vet Med Assoc (1992) 200(6):829–34. 1568933

[36] Gamlem H Nordstoga K Arnesen K. Canine vascular neoplasia–a population-based clinicopathologic study of 439 tumours and tumour-like lesions in 420 dogs. APMIS Suppl (2008) 116(125):41–54. doi: 10.1111/j.1600-0463.2008.125m4.x 19385280

[37] Schultheiss PC. A retrospective study of visceral and nonvisceral hemangiosarcoma and hemangiomas in domestic animals. J Vet Diagn Invest (2004) 16(6):522–6. doi: 10.1177/104063870401600606 15586567

[38] Kent MS Burton JH Dank G Bannasch DL Rebhun RB. Association of cancer-related mortality, age and gonadectomy in golden retriever dogs at a veterinary academic center (1989-2016). PloS One (2018) 13(2):e0192578. doi: 10.1371/journal.pone.0192578 29408871 PMC5800597

[39] Stewart FW Treves N. Lymphangiosarcoma in postmastectomy lymphedema; a report of six cases in Elephantiasis chirurgica. Cancer (1948) 1(1):64–81. doi: 10.1002/1097-0142(194805)1:1<64::aid-cncr2820010105>3.0.co;2-w 18867440

[40] Huang J Mackillop WJ. Increased risk of soft tissue sarcoma after radiotherapy in women with breast carcinoma. Cancer (2001) 92(1):172–80. doi: 10.1002/1097-0142(20010701)92:1<172::aid-cncr1306>3.0.co;2-k 11443624

[41] West JG Weitzel JN Tao ML Carpenter M West JE Fanning C. Brca mutations and the risk of angiosarcoma after breast cancer treatment. Clin Breast Cancer (2008) 8(6):533–7. doi: 10.3816/CBC.2008.n.066 19073510

[42] Bosetti C La Vecchia C Lipworth L McLaughlin JK. Occupational exposure to vinyl chloride and cancer risk: A review of the epidemiologic literature. Eur J Cancer Prev (2003) 12(5):427–30. doi: 10.1097/00008469-200310000-00012 14512808

[43] Ron E. Cancer risks from medical radiation. Health Phys (2003) 85(1):47–59. doi: 10.1097/00004032-200307000-00011 12852471

[44] Locker GY Doroshow JH Zwelling LA Chabner BA. The clinical features of hepatic angiosarcoma: A report of four cases and a review of the English literature. Med (Baltimore) (1979) 58(1):48–64. doi: 10.1097/00005792-197901000-00003 368508

[45] Ben-Izhak O Kerner H Brenner B Lichtig C. Angiosarcoma of the colon developing in a capsule of a foreign body. Report of a case with associated hemorrhagic diathesis. Am J Clin Pathol (1992) 97(3):416–20. doi: 10.1093/ajcp/97.3.416 1543166

[46] Weiss WM Riles TS Gouge TH Mizrachi HH. Angiosarcoma at the site of a dacron vascular prosthesis: A case report and literature review. J Vasc Surg (1991) 14(1):87–91. doi: 10.1016/0741-5214(91)90158-q 1829490

[47] McDonald DJ Enneking WF Sundaram M. Metal-associated angiosarcoma of bone: report of two cases and review of the literature. Clin Orthop Relat Res (2002) 396):206–14. doi: 10.1097/00003086-200203000-00031 11859245

[48] Folpe AL Johnston CA Weiss SW. Cutaneous angiosarcoma arising in a gouty tophus: report of a unique case and a review of foreign material-associated angiosarcomas. Am J Dermatopathol (2000) 22(5):418–21. doi: 10.1097/00000372-200010000-00006 11048977

[49] Goedert JJ Cote TR Virgo P Scoppa SM Kingma DW Gail MH. Spectrum of aids-associated Malignant disorders. Lancet (1998) 351(9119):1833–9. doi: 10.1016/s0140-6736(97)09028-4 9652666

[50] Tonomura N Elvers I Thomas R Megquier K Turner-Maier J Howald C. Genome-wide association study identifies shared risk loci common to two Malignancies in golden retrievers. PloS Genet (2015) 11(2):e1004922. doi: 10.1371/journal.pgen.1004922 25642983 PMC4333733

[51] Hargis AM Ihrke PJ Spangler WL Stannard AA. A retrospective clinicopathologic study of 212 dogs with Cutaneous hemangiomas and hemangiosarcomas. Vet Pathol (1992) 29(4):316–28. doi: 10.1177/030098589202900406 1514218

[52] Hargis AM Lee AC Thomassen RW. Tumor and tumor-like lesions of perilimbal conjunctiva in laboratory dogs. J Am Vet Med Assoc (1978) 173(9):1185–90. 570186

[53] Wong K Ludwig L Krijgsman O Adams DJ Wood GA van der Weyden L. Comparison of the oncogenomic landscape of canine and feline hemangiosarcoma shows novel parallels with human angiosarcoma. Dis Model Mech (2021) 14(7):dmm049044. doi: 10.1242/dmm.049044 34296746 PMC8319545

[54] Fletcher CD. Diagnostic Histopathology of Tumors. Oxford, UK: Elsevier Health Sciences (2007).

[55] Fletcher C Bridge JA Hogendoorn PCW Mertens F. Who Classification of Tumours of Soft Tissue and Bone: WHO Classification of Tumours Vol. vol. 5. Geneva, Switzerland: World Health Organization (2013).

[56] Ohsawa M Naka N Tomita Y Kawamori D Kanno H Aozasa K. Use of immunohistochemical procedures in diagnosing angiosarcoma. Evaluation of 98 Cases. Cancer (1995) 75(12):2867–74. doi: 10.1002/1097-0142(19950615)75:12<2867::aid-cncr2820751212>3.0.co;2-8 7773935

[57] von Beust BR Suter MM Summers BA. Factor Viii-related antigen in canine endothelial neoplasms: an immunohistochemical study. Vet Pathol (1988) 25(4):251–5. doi: 10.1177/030098588802500401 3136585

[58] Ferrer L Fondevila D Rabanal RM Vilafranca M. Immunohistochemical detection of Cd31 antigen in normal and neoplastic canine endothelial cells. J Comp Pathol (1995) 112(4):319–26. doi: 10.1016/s0021-9975(05)80013-1 7593754

[59] Rozolen JM Teodoro TGW Sobral RA Sueiro FAR Laufer-Amorim R Elias F. Investigation of prognostic value of claudin-5, Psma, and Ki67 expression in canine splenic hemangiosarcoma. Anim (Basel) (2021) 11(8):2406. doi: 10.3390/ani11082406 PMC8388721 34438863

[60] Sabattini S Bettini G. An immunohistochemical analysis of canine haemangioma and haemangiosarcoma. J Comp Pathol (2009) 140(2-3):158–68. doi: 10.1016/j.jcpa.2008.10.006 19091326

[61] Campos AG Campos JADB Sanches DS Dagli MLZ Matera JM. Immunohistochemical evaluation of vascular endothelial growth factor (Vegf) in splenic hemangiomas and hemangiosarcomas in dogs. Open J Vet Med (2012) 2(4):191–5. doi: 10.4236/ojvm.2012.24030

[62] Gorden BH Kim J-H Sarver AL Frantz AM Breen M Lindblad-Toh K. Identification of three molecular and functional subtypes in canine hemangiosarcoma through gene expression profiling and progenitor cell characterization. Am J Pathol (2014) 184(4):985–95. doi: 10.1016/j.ajpath.2013.12.025 PMC3969990 24525151

[63] Lamerato-Kozicki AR Helm KM Jubala CM Cutter GC Modiano JF. Canine hemangiosarcoma originates from hematopoietic precursors with potential for endothelial differentiation. Exp Hematol (2006) 34(7):870–8. doi: 10.1016/j.exphem.2006.04.013 16797414

[64] Painter CA Jain E Tomson BN Dunphy M Stoddard RE Thomas BS. The angiosarcoma project: enabling genomic and clinical discoveries in a rare cancer through patient-partnered research. Nat Med (2020) 26(2):181–7. doi: 10.1038/s41591-019-0749-z 32042194

[65] Murali R Chandramohan R Moller I Scholz SL Berger M Huberman K. Targeted massively parallel sequencing of angiosarcomas reveals frequent activation of the mitogen activated protein kinase pathway. Oncotarget (2015) 6(34):36041–52. doi: 10.18632/oncotarget.5936 PMC4742160 26440310

[66] Megquier K Turner-Maier J Swofford R Kim JH Sarver AL Wang C. Comparative genomics reveals shared mutational landscape in canine hemangiosarcoma and human angiosarcoma. Mol Cancer Res (2019) 17(12):2410–21. doi: 10.1158/1541-7786.MCR-19-0221 PMC7067513 31570656

[67] Naka N Tomita Y Nakanishi H Araki N Hongyo T Ochi T. Mutations of P53 tumor-suppressor gene in angiosarcoma. Int J Cancer (1997) 71(6):952–5. doi: 10.1002/(sici)1097-0215(19970611)71:6<952::aid-ijc7>3.0.co;2-x 9185695

[68] Wang G Wu M Durham AC Radaelli E Mason NJ Xu X. Molecular subtypes in canine hemangiosarcoma reveal similarities with human angiosarcoma. PloS One (2020) 15(3):e0229728. doi: 10.1371/journal.pone.0229728 32210430 PMC7094861

[69] Weihrauch M Bader M Lehnert G Koch B Wittekind C Wrbitzky R. Mutation analysis of K-Ras-2 in liver angiosarcoma and adjacent nonneoplastic liver tissue from patients occupationally exposed to vinyl chloride. Environ Mol Mutagen (2002) 40(1):36–40. doi: 10.1002/em.10084 12211074

[70] Behjati S Tarpey PS Sheldon H Martincorena I Van Loo P Gundem G. Recurrent Ptprb and Plcg1 mutations in angiosarcoma. Nat Genet (2014) 46(4):376–9. doi: 10.1038/ng.2921 PMC4032873 24633157

[71] Wang G Wu M Maloneyhuss MA Wojcik J Durham AC Mason NJ. Actionable mutations in canine hemangiosarcoma. PloS One (2017) 12(11):e0188667. doi: 10.1371/journal.pone.0188667 29190660 PMC5708669

[72] Dim D Ravi V Tan J Hicks D Wong M. The actin-bundling motility protein fascin and vascular endothelial growth factor (Vegf) are universally over-expressed in human angiosarcoma. J Clin Oncol (2007) 25(18_suppl):10068–. doi: 10.1200/jco.2007.25.18_suppl.10068

[73] Zietz C Rossle M Haas C Sendelhofert A Hirschmann A Sturzl M. Mdm-2 oncoprotein overexpression, P53 gene mutation, and Vegf up-regulation in angiosarcomas. Am J Pathol (1998) 153(5):1425–33. doi: 10.1016/S0002-9440(10)65729-X PMC1876718 9811333

[74] Garcia JM Gonzalez R Silva JM Dominguez G Vegazo IS Gamallo C. Mutational status of K-Ras and Tp53 genes in primary sarcomas of the heart. Br J Cancer (2000) 82(6):1183–5. doi: 10.1054/bjoc.1999.1060 PMC2363343 10735503

[75] Wagner MJ Ravi V Menter DG Sood AK. Endothelial cell Malignancies: new insights from the laboratory and clinic. NPJ Precis Oncol (2017) 1(1):11. doi: 10.1038/s41698-017-0013-2 29872699 PMC5859470

[76] Ueda T Oji Y Naka N Nakano Y Takahashi E Koga S. Overexpression of the Wilms' Tumor gene Wt1 in human bone and soft-tissue sarcomas. Cancer Sci (2003) 94(3):271–6. doi: 10.1111/j.1349-7006.2003.tb01432.x PMC11160304 12824921

[77] Johnson KD Glinskii OV Mossine VV Turk JR Mawhinney TP Anthony DC. Galectin-3 as a potential therapeutic target in tumors arising from Malignant endothelia. Neoplasia (2007) 9(8):662–70. doi: 10.1593/neo.07433 PMC1950436 17786185

[78] Naito S Shimizu K Nakashima M Nakayama T Ito T Ito M. Overexpression of Ets-1 transcription factor in angiosarcoma of the skin. Pathol Res Pract (2000) 196(2):103–9. doi: 10.1016/S0344-0338(00)80041-2 10707367

[79] Dictor M Bendsoe N Runke S Witte M. Major basement membrane components in Kaposi's sarcoma, angiosarcoma and benign vascular neogenesis. J Cutan Pathol (1995) 22(5):435–41. doi: 10.1111/j.1600-0560.1995.tb00759.x 8594076

[80] Miettinen M Sarlomo-Rikala M Lasota J. Kit expression in angiosarcomas and fetal endothelial cells: lack of mutations of exon 11 and exon 17 of C-Kit. Mod Pathol (2000) 13(5):536–41. doi: 10.1038/modpathol.3880093 10824925

[81] Hornick JL Fletcher CD. Immunohistochemical staining for Kit (Cd117) in soft tissue sarcomas is very limited in distribution. Am J Clin Pathol (2002) 117(2):188–93. doi: 10.1309/LX9U-F7P0-UWDH-8Y6R 11865845

[82] Komdeur R Hoekstra HJ Molenaar WM Van Den Berg E Zwart N Pras E. Clinicopathologic assessment of postradiation sarcomas: kit as a potential treatment target. Clin Cancer Res (2003) 9(8):2926–32. 12912938

[83] Itakura E Yamamoto H Oda Y Tsuneyoshi M. Detection and characterization of vascular endothelial growth factors and their receptors in a series of angiosarcomas. J Surg Oncol (2008) 97(1):74–81. doi: 10.1002/jso.20766 18041747

[84] Agulnik M Yarber JL Okuno SH von Mehren M Jovanovic BD Brockstein BE. An open-label, multicenter, phase II study of bevacizumab for the treatment of angiosarcoma and epithelioid hemangioendotheliomas. Ann Oncol (2013) 24(1):257–63. doi: 10.1093/annonc/mds237 22910841

[85] Ray-Coquard IL Domont J Tresch-Bruneel E Bompas E Cassier PA Mir O. Paclitaxel given once per week with or without bevacizumab in patients with advanced angiosarcoma: A randomized phase II trial. J Clin Oncol (2015) 33(25):2797–802. doi: 10.1200/JCO.2015.60.8505 26215950

[86] Arbiser JL Moses MA Fernandez CA Ghiso N Cao Y Klauber N. Oncogenic H-Ras stimulates tumor angiogenesis by two distinct pathways. Proc Natl Acad Sci U.S.A. (1997) 94(3):861–6. doi: 10.1073/pnas.94.3.861 PMC19604 9023347

[87] LaMontagne KR Jr. Moses MA Wiederschain D Mahajan S Holden J Ghazizadeh H. Inhibition of map kinase kinase causes morphological reversion and dissociation between soft agar growth and in vivo tumorigenesis in angiosarcoma cells. Am J Pathol (2000) 157(6):1937–45. doi: 10.1016/s0002-9440(10)64832-8 PMC1885752 11106566

[88] Dickerson EB Thomas R Fosmire SP Lamerato-Kozicki AR Bianco SR Wojcieszyn JW. Mutations of phosphatase and tensin homolog deleted from chromosome 10 in canine hemangiosarcoma. Vet Pathol (2005) 42(5):618–32. doi: 10.1354/vp.42-5-618 16145208

[89] Thomas R Borst L Rotroff D Motsinger-Reif A Lindblad-Toh K Modiano JF. Genomic profiling reveals extensive heterogeneity in somatic DNA copy number aberrations of canine hemangiosarcoma. Chromosome Res (2014) 22(3):305–19. doi: 10.1007/s10577-014-9406-z PMC5518683 24599718

[90] Gardner HL London CA Portela RA Nguyen S Rosenberg MP Klein MK. Maintenance therapy with toceranib following doxorubicin-based chemotherapy for canine splenic hemangiosarcoma. BMC Vet Res (2015) 11(1):131. doi: 10.1186/s12917-015-0446-1 26062540 PMC4464614

[91] Abraham JA Hornicek FJ Kaufman AM Harmon DC Springfield DS Raskin KA. Treatment and outcome of 82 patients with angiosarcoma. Ann Surg Oncol (2007) 14(6):1953–67. doi: 10.1245/s10434-006-9335-y 17356953

[92] Fayette J Martin E Piperno-Neumann S Le Cesne A Robert C Bonvalot S. Angiosarcomas, a heterogeneous group of sarcomas with specific behavior depending on primary site: A retrospective study of 161 cases. Ann Oncol (2007) 18(12):2030–6. doi: 10.1093/annonc/mdm381 17974557

[93] Pawlik TM Paulino AF McGinn CJ Baker LH Cohen DS Morris JS. Cutaneous angiosarcoma of the scalp: A multidisciplinary approach. Cancer (2003) 98(8):1716–26. doi: 10.1002/cncr.11667 14534889

[94] Heinhuis KM IJ NS van der Graaf WTA Kerst JM Schrage Y Beijnen JH. Neoadjuvant systemic treatment of primary angiosarcoma. Cancers (Basel) (2020) 12(8):2251. doi: 10.3390/cancers12082251 32806524 PMC7464310

[95] Young RJ Natukunda A Litiere S Woll PJ Wardelmann E van der Graaf WT. First-line anthracycline-based chemotherapy for angiosarcoma and other soft tissue sarcoma subtypes: pooled analysis of eleven European organisation for research and treatment of cancer soft tissue and bone sarcoma group trials. Eur J Cancer (2014) 50(18):3178–86. doi: 10.1016/j.ejca.2014.10.004 25459395

[96] Spangler WL Kass PH. Pathologic factors affecting postsplenectomy survival in dogs. J Vet Intern Med (1997) 11(3):166–71. doi: 10.1111/j.1939-1676.1997.tb00085.x 9183768

[97] Yamamoto S Hoshi K Hirakawa A Chimura S Kobayashi M Machida N. Epidemiological, clinical and pathological features of primary cardiac hemangiosarcoma in dogs: A review of 51 cases. J Vet Med Sci (2013) 75(11):1433–41. doi: 10.1292/jvms.13-0064 PMC3942993 23811814

[98] Weisse C Soares N Beal MW Steffey MA Drobatz KJ Henry CJ. Survival times in dogs with right atrial hemangiosarcoma treated by means of surgical resection with or without adjuvant chemotherapy: 23 cases (1986–2000). J Am Vet Med (2005) 226(4):575–9. doi: 10.2460/javma.2005.226.575 15742699

[99] Hillers KR Lana SE Fuller CR LaRue SM. Effects of palliative radiation therapy on nonsplenic hemangiosarcoma in dogs. J Am Anim Hosp Assoc (2007) 43(4):187–92. doi: 10.5326/0430187 17615398

[100] Koontz BF Miles EF Rubio MA Madden JF Fisher SR Scher RL. Preoperative radiotherapy and bevacizumab for angiosarcoma of the head and neck: two case studies. Head Neck (2008) 30(2):262–6. doi: 10.1002/hed.20674 17685450

[101] Verschraegen CF Fekrazad HM Rabinowitz I Quinn R Snyder D Judson P. Phase I/II study of docetaxel (D), gemcitabine (G), and bevacizumab (B) in patients (Pts) with advanced or recurrent soft tissue sarcoma (Sts). J Clin Oncol (2007) 25(18_suppl):10056–. doi: 10.1200/jco.2007.25.18_suppl.10056

[102] Kollar A Jones RL Stacchiotti S Gelderblom H Guida M Grignani G. Pazopanib in advanced vascular sarcomas: an Eortc soft tissue and bone sarcoma group (Stbsg) retrospective analysis. Acta Oncol (2017) 56(1):88–92. doi: 10.1080/0284186X.2016.1234068 27838944

[103] Marabelle A Fakih M Lopez J Shah M Shapira-Frommer R Nakagawa K. Association of tumour mutational burden with outcomes in patients with advanced solid tumours treated with pembrolizumab: prospective biomarker analysis of the multicohort, open-label, phase 2 keynote-158 study. Lancet Oncol (2020) 21(10):1353–65. doi: 10.1016/S1470-2045(20)30445-9 32919526

[104] Khanna C Rosenberg M Vail D. A review of paclitaxel and novel formulations including those suitable for use in dogs. J Vet Intern Med (2015) 29(4):1006–12. doi: 10.1111/jvim.12596 PMC4895360 26179168

[105] Maekawa N Konnai S Takagi S Kagawa Y Okagawa T Nishimori A. A canine chimeric monoclonal antibody targeting Pd-L1 and its clinical efficacy in canine oral Malignant melanoma or undifferentiated sarcoma. Sci Rep (2017) 7(1):8951. doi: 10.1038/s41598-017-09444-2 28827658 PMC5567082

[106] Igase M Nemoto Y Itamoto K Tani K Nakaichi M Sakurai M. A pilot clinical study of the therapeutic antibody against canine Pd-1 for advanced spontaneous cancers in dogs. Sci Rep (2020) 10(1):18311. doi: 10.1038/s41598-020-75533-4 33110170 PMC7591904

[107] Kim SE Liptak JM Gall TT Monteith GJ Woods JP. Epirubicin in the adjuvant treatment of splenic hemangiosarcoma in dogs: 59 cases (1997-2004). J Am Vet Med Assoc (2007) 231(10):1550–7. doi: 10.2460/javma.231.10.1550 18021000

[108] Sorenmo K Samluk M Clifford C Baez J Barrett JS Poppenga R. Clinical and pharmacokinetic characteristics of intracavitary administration of pegylated liposomal encapsulated doxorubicin in dogs with splenic hemangiosarcoma. J Vet Intern Med (2007) 21(6):1347–54. doi: 10.1892/06-214.1 18196746

[109] Kahn SA Mullin CM de Lorimier LP Burgess KE Risbon RE Fred RM 3rd. Doxorubicin and deracoxib adjuvant therapy for canine splenic hemangiosarcoma: a pilot study. Can Vet J (2013) 54(3):237–42. PMC3573628 23997259

[110] Bray J Orbell G Cave N Munday J. Does thalidomide prolong survival in dogs with splenic haemangiosarcoma? J Small Anim Pract (2018) 59(2):85–91. doi: 10.1111/jsap.12796 29210452

[111] Rowinsky EK. The development and clinical utility of the taxane class of antimicrotubule chemotherapy agents. Annu Rev Med (1997) 48(1):353–74. doi: 10.1146/annurev.med.48.1.353 9046968

[112] Poirier VJ Hershey AE Burgess KE Phillips B Turek MM Forrest LJ. Efficacy and toxicity of paclitaxel (Taxol) for the treatment of canine Malignant tumors. J Vet Intern Med (2004) 18(2):219–22. doi: 10.1892/0891-6640(2004)18<219:eatopt>2.0.co 15058774

[113] U'Ren LW Biller BJ Elmslie RE Thamm DH Dow SW. Evaluation of a novel tumor vaccine in dogs with hemangiosarcoma. J Vet Intern Med (2007) 21(1):113–20. doi: 10.1892/0891-6640(2007)21[113:eoantv]2.0.co;2 17338158

[114] Vail DM MacEwen EG Kurzman ID Dubielzig RR Helfand SC Kisseberth WC. Liposome-encapsulated muramyl tripeptide phosphatidylethanolamine adjuvant immunotherapy for splenic hemangiosarcoma in the dog: a randomized multi-institutional clinical trial. Clin Cancer Res (1995) 1(10):1165–70. 9815908

[115] Brown DC Reetz J. Single agent polysaccharopeptide delays metastases and improves survival in naturally occurring hemangiosarcoma. Evid Based Complement Alternat Med (2012) 2012:384301. doi: 10.1155/2012/384301 22988473 PMC3440946

[116] Masuzawa M Fujimura T Hamada Y Fujita Y Hara H Nishiyama S. Establishment of a human hemangiosarcoma cell line (Iso-Has). Int J Cancer (1999) 81(2):305–8. doi: 10.1002/(sici)1097-0215(19990412)81:2<305::aid-ijc22>3.0.co;2-z 10188735

[117] Krump-Konvalinkova V Bittinger F Olert J Brauninger W Brunner J Kirkpatrick CJ. Establishment and characterization of an angiosarcoma-derived cell line, as-M. Endothelium (2003) 10(6):319–28. doi: 10.1080/10623320390272316 14741847

[118] Versleijen-Jonkers YMH Hillebrandt-Roeffen MHS Weidema ME Mooren J von Rhein DT de Bitter TJJ. Establishment and characterization of the first patient-derived radiation-induced angiosarcoma xenograft model (Rt-As5). Sci Rep (2023) 13(1):2653. doi: 10.1038/s41598-023-29569-x 36788310 PMC9929321

[119] Yates N Hinkel J. The Economics of moonshots: value in rare disease drug development. Clin Transl Sci (2022) 15(4):809–12. doi: 10.1111/cts.13270 PMC9010265 35334152

[120] Thamm DH Dickerson EB Akhtar N Lewis R Auerbach R Helfand SC. Biological and molecular characterization of a canine hemangiosarcoma-derived cell line. Res Vet Sci (2006) 81(1):76–86. doi: 10.1016/j.rvsc.2005.09.005 16256156

[121] Fosmire SP Dickerson EB Scott AM Bianco SR Pettengill MJ Meylemans H. Canine Malignant hemangiosarcoma as a model of primitive angiogenic endothelium. Lab Invest (2004) 84(5):562–72. doi: 10.1038/labinvest.3700080 15064773

[122] Murai A Asa SA Kodama A Hirata A Yanai T Sakai H. Constitutive phosphorylation of the Mtorc2/Akt/4e-Bp1 pathway in newly derived canine hemangiosarcoma cell lines. BMC Vet Res (2012) 8:128. doi: 10.1186/1746-6148-8-128 22839755 PMC3438112

[123] Urbasic AS Hynes S Somrak A Contakos S Rahman MM Liu J. Oncolysis of canine tumor cells by myxoma virus lacking the Serp2 gene. Am J Vet Res (2012) 73(8):1252–61. doi: 10.2460/ajvr.73.8.1252 22849686

[124] Kim JH Frantz AM Anderson KL Graef AJ Scott MC Robinson S. Interleukin-8 promotes canine hemangiosarcoma growth by regulating the tumor microenvironment. Exp Cell Res (2014) 323(1):155–64. doi: 10.1016/j.yexcr.2014.02.020 PMC4256199 24582862

[125] Tamburini BA Trapp S Phang TL Schappa JT Hunter LE Modiano JF. Gene expression profiles of sporadic canine hemangiosarcoma are uniquely associated with breed. PloS One (2009) 4(5):e5549. doi: 10.1371/journal.pone.0005549 19461996 PMC2680013

[126] Cook D Brown D Alexander R March R Morgan P Satterthwaite G. Lessons learned from the fate of Astrazeneca's drug pipeline: A five-dimensional framework. Nat Rev Drug Discovery (2014) 13(6):419–31. doi: 10.1038/nrd4309 24833294

[127] Blair HA. Onasemnogene Abeparvovec: A review in spinal muscular atrophy. CNS Drugs (2022) 36(9):995–1005. doi: 10.1007/s40263-022-00941-1 35960489

[128] Miller T Cudkowicz M Shaw PJ Andersen PM Atassi N Bucelli RC. Phase 1-2 trial of antisense oligonucleotide tofersen for Sod1 Als. N Engl J Med (2020) 383(2):109–19. doi: 10.1056/NEJMoa2003715 32640130

[129] International conference on harmonisation of technical requirements for registration of pharmaceuticals for human use. Preclinical safety evaluation of biotechnology-derived pharmaceuticals S6(R1). In: ICH Harmonised Tripartite Guideline. Geneva, Switzerland: ICH Expert Working Group (2011).

